# Development of Polyvinyl Alcohol Hydrogels for Controlled Glucose Release in Biomedical Applications

**DOI:** 10.3390/gels10100668

**Published:** 2024-10-19

**Authors:** Rosa M. Quispe-Siccha, Osvaldo I. Medina-Sandoval, Abraham Estrada-Tinoco, Jorge A. Pedroza-Pérez, Adolfo Martínez-Tovar, Irma Olarte-Carrillo, Rafael Cerón-Maldonado, Arturo Reding-Bernal, Juan C. López-Alvarenga

**Affiliations:** 1 Research and Technological Development Unit, Research Department, General Hospital of Mexico, “Dr. Eduardo Liceaga”, Mexico City 06726, Mexico; 2 Interdisciplinary Biotechnology Professional Unit, National Polytechnic Institute, Mexico City 07340, Mexico; oimsipn@gmail.com (O.I.M.-S.); abraham_et_ma@live.com.mx (A.E.-T.); alan.japp3@gmail.com (J.A.P.-P.); 3 Hematology Laboratory, General Hospital of Mexico “Dr. Eduardo Liceaga”, Mexico City 06726, Mexico; mtadolfo@gmail.com (A.M.-T.); irma.olarte@salud.gob.mx (I.O.-C.); cmrafael.bh@gmail.com (R.C.-M.); 4 Research Department, General Hospital of Mexico “Dr. Eduardo Liceaga”, Mexico City 06726, Mexico; arturo.reding@salud.gob.mx; 5 Population Health & Biostatistics, School of Medicine, University of Texas Rio Grande Valley, Edinburgh, TX 78539, USA; juan.lopezalvarenga@utrgv.edu

**Keywords:** polyvinyl alcohol hydrogel, dehydration, storage capacity, thermal resistance, permeability, glucose release, diffusion speed, flow, cell growth

## Abstract

Polyvinyl alcohol (PVA) hydrogels have a wide range of applications in the pharmaceutical and biomedicine fields due to their exceptional biophysical properties. The study focuses on preparing and characterizing capsule-shaped PVA hydrogels to enhance their biocompatibility and porosity for controlled glucose release and cell proliferation. The hydrogels were prepared using different concentrations (Cs) and molecular weights (MWs) of PVA, with two different lengths, A (10 mm) and B (20 mm), to control glucose release over 60 min. The preparation process involved PVA gel preparation and PVA hydrogel formation. A total of 500 µL of glucose was injected into all dehydrated hydrogels in groups A and B. Glucose release was studied by immersing the hydrogels in saline at 37 °C with stirring at 500 rpm. The SUP-B15 cell line was grown in six A1 hydrogels for biocompatibility testing. The results indicate that all hydrogels remained stable at 37 °C without degrading. Those with a higher C and MW exhibited a denser and less porous structure, lower glucose storage capacity, and higher elongation at break. Significant differences in glucose release, diffusion speed, and flux were observed, which were more evident in A1 > A4, B1 > B4, and B1 > A1 over 60 min. A1 and B1 had higher values because their higher porosity distribution allowed glucose to diffuse more easily. B1, being larger, has more glucose due to its increased length. The cell growth response and viability at 48 h in contact with the hydrogels was similar to that of the control (4.5 × 10^5^ cells/mL, 98.5% vs. 4.8 × 10^5^ cells/mL, 99.7% viability), thus demonstrating biocompatibility. The hydrogels effectively released glucose over 60 min, with variations based on porosity, C, MW, and length, and demonstrated good biocompatibility with the cell line.

## 1. Introduction

Polyvinyl alcohol (PVA) hydrogels exhibit various physical and chemical properties that significantly influence drug release, making them versatile for multiple biomedical applications. The freeze–thaw (F/T) method commonly used to prepare PVA hydrogels affects their swelling ability and stability, affecting drug release [1]. For example, hydrogels with fewer F/T cycles and higher freezing temperatures show higher swelling rates, which improves their ability to release drugs over extended periods [1].

Mechanical properties such as tensile stress and Young’s modulus are also critical; for example, PVA hydrogels subjected to stretching cycles between F/T cycles exhibit higher crystallinity and stiffness, which can improve the drug release rate [2]. The molecular weight and concentration of PVA further influence the hydrogel’s ability to absorb and transfer energy, affecting the release kinetics of the encapsulated drugs [3].

The elongation at the break of PVA hydrogels is a crucial aspect of their characterization because it measures their ductility or ability to deform plastically before breaking [3]. This is important in applications requiring flexibility and adaptability, such as dressings or implants [4]. Additionally, the storage modulus, known as Young’s modulus (G′), and the loss modulus (G″) are important for characterizing viscoelastic materials like PVA hydrogels and composites with other materials [2,5,6,7]. G′ describes a material’s stiffness or elasticity, while G″ is associated with damping or viscoelastic behavior.

The storage capacity of substances in a PVA hydrogel depends on its porous structure and ability to absorb water. PVA hydrogels are highly porous materials with a great capacity to retain water. The pores in PVA hydrogels act as reservoirs, absorbing and holding liquids, including water and dissolved substances [1,3]. This property is crucial for controlled drug release or tissue engineering [8,9,10]. Additionally, PVA hydrogels are known for their excellent thermal stability compared to other materials, providing greater resistance to degradation when exposed to temperature changes [11,12]. Their thermal resistance allows them to be used in environments with temperature fluctuations, such as dressings or controlled drug-release systems and tissue engineering [8,13,14].

The concentration of PVA is crucial for determining the distribution of porosity in hydrogels [2,15,16] and their degree of swelling [1,3,17]. By adjusting the concentration and optimizing cross-linking, their properties can be controlled for specific applications. For instance, it can change the release rate of substances in controlled-release applications according to therapeutic needs [8,18]. In tissue regeneration, PVA hydrogels serve as scaffolds for cell growth, promoting cell adhesion and proliferation in a three-dimensional environment through the diffusion of nutrients and oxygen [3,19,20,21,22,23]. These properties are also vital in applications such as medical dressings, which aim to keep a wound moist to facilitate healing [4,9,14,24]. Additionally, PVA hydrogels are used in sensors and actuators, such as pH or glucose sensors [25,26,27].

PVA is more biomimetic compared to other synthetic polymers and can be used as matrices in tissue engineering applications, as well as a vehicle for controlled drug release [8,20,28,29]. Additionally, PVA hydrogels can create scaffolds capable of housing various types of cells, such as pancreatic and corneal cells. This facilitates the implantation of cells to carry out their functions without triggering adverse reactions from the transplantation [3,30]. For instance, a bioartificial pancreas can shield transplanted islets from the recipient’s immune response by enveloping them with a semipermeable membrane or hydrogel barrier. The bioartificial pancreas prevents immunocompetent cells, antibodies, or complement from breaching the semipermeable barrier, ultimately providing immunoprotecting. Furthermore, the device can respond to blood glucose levels by releasing insulin [19,20].

Blending PVA with other polymers can enhance its mechanical properties. When combined with chitosan (CT), PVA can improve hydrogels’ tensile strength, flexibility, bulk, and surface hydrophilicity [31]. For instance, incorporating CT into PVA hydrogels results in a more homogeneous network structure, enhancing mechanical stability, reducing porosity, and being more bioabsorbable. This is crucial for maintaining cell viability during preservation [32,33]. Additionally, when PVA is mixed with polyethylene glycol (PEG), it can achieve controlled drug release. A higher PEG content leads to the reduced swelling of hydrogels [8]. Moreover, modifying PVA with cellulose powder and poly (ethylene glycol) (PEG) has been shown to enhance the hydrogels’ biocompatibility and cytocompatibility, making them suitable for cell growth and tissue engineering applications [34]. Moreover, uniform PVA microparticles can be utilized to microencapsulate drugs through membrane emulsification and chemical cross-linking, allowing a higher drug content storage [14,35]. PVA mixed with Ag nanoparticles has an improved antibacterial effect for wound dressing applications [14]. However, in some cases, blending PVA with other polymers can be counterproductive and even toxic, especially when chemically induced cross-linking occurs [36].

The combined physical and chemical properties, such as swelling behavior, mechanical strength, porosity, and presence of additional materials, determine the effectiveness and rate of substance release from PVA hydrogels. This makes them well-suited for specific drug delivery systems, cell preservation, and survival, and effective as scaffolds. In this study, we constructed and characterized PVA hydrogels as capsules of two lengths. We varied their concentration (C) and molecular weight (MW) to examine their porosity distribution (pores number versus diameter) and to assess their release behavior from the inside to the outside of the hydrogel, diffusion speed, and diffusion flow of glucose in a time interval of 60 min. The hydrogels were physically cross-linked using freeze/thaw techniques, eliminating the need for toxic chemicals and mixing with other polymers, ensuring biocompatibility and immunoprotection [36]. Based on this study’s findings, combining them with different polymers is possible in future studies. This study developed a method for producing hydrogels that can release substances, such as glucose, from the interior to the exterior of a hydrogel within 60 min. These hydrogels have been shown to be biocompatible with the SUP-B15 cell line, supporting their preservation and proliferation. This presents new possibilities for future research involving other cell lines to assess their preservation and cell viability through a nutrient exchange.

## 2. Results and Discussion

This section presents the results obtained from the PVA hydrogels of Groups A and B. Group A comprises hydrogels with a length of 10 mm, while Group B consists of hydrogels with a length of 20 mm. The formulations for these groups are as follows: A1 and B1 (C1, MW1); A2 and B2 (C2, MW1); A3 and B3 (C1, MW2); and A4 and B4 (C2, MW2), as detailed in the methodology.

### 2.1. Dehydration

Table 1 shows the percentage of water mass lost (*W_L_*) as a function of C and MW for the A and B group hydrogels due to dehydration. The results are an average of 4 hydrogels for each experiment, which is 32 hydrogels.

The purpose of dehydrating the hydrogels was to increase their storage capacity for glucose. The dehydration time was carefully chosen for each group to prevent their original length and shape from being compromised when injected with glucose. Group B hydrogels lost more water mass than Group A hydrogels because they were larger. Hydrogels with higher C and MW tend to experience lower water mass loss due to a greater porosity distribution but smaller pore size. These results are related to the following studies [37].

The dehydration percentage of our hydrogels was kept low compared to other studies to preserve the original structure when rehydrating them with glucose filling [37,38]. Dehydrated hydrogels can be utilized for controlled drug release by slowly rehydrating them to enable sustained drug release [37]. In biomedical applications, dehydrated hydrogels can be used for cell preservation and transport, being rehydrated at the time of use [23].

### 2.2. Swelling Ratio

The data presented in Table 2 show that the hydrogels’ swelling ratio ($W_{s}$) varies depending on the C and MW of PVA. The results indicate that the $W_{s}$ decreases as the C and MW increase. This trend is observed in both groups of hydrogels A and B, with group B exhibiting a larger $W_{s}$ due to its larger size. The results represent an average of four hydrogels for each PVA hydrogel formulation.

The distribution and interconnection of pores in a material are mainly influenced by the composition of the polymer chains and the cross-linking density. When the polymer concentration in a given volume increases, the number of organized polymer chains increases, leading to decreased porosity within the material. High MW polymers typically have a finer pore structure due to longer and cross-linked chains, which may explain their reduced water retention capacity [3,37].

Hydrogel swelling is a crucial characteristic that can be controlled by adjusting the C and MW of PVA, the freeze–thaw time, and the number of freeze–thaw cycles. By regulating hydrogel swelling, it is possible to use them as controlled release systems and create a favorable environment for cells, enabling cell adhesion [3].

### 2.3. Storage Capacity and Thermal Resistance

**Figure 1:**
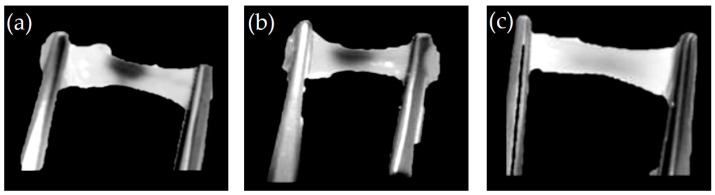
The hydrogel at different stages of exposure to temperature and stirring. The hydrogel is in its original state before being subjected to heat and stirring (**a**), the hydrogel was exposed to 37 °C and constant stirring of 500 rpm for 20 min (**b**), and the hydrogel after being exposed to 37 °C and continuous stirring at 500 rpm for 30 min (**c**).

The storage capacity of the milk–green dye mixture within the A1 hydrogels averaged 430 ± 12 μL, and for the B1 hydrogels, it was 450 ± 10 μL. Additionally, both hydrogels demonstrated thermal resistance at 37 °C with a constant rotation of 500 rpm without deformation, as shown in Figure 1. When stretched, they did not break. Furthermore, the mixture decreased after three intervals: the original state, 20 min, and 30 min.

The experiment was conducted on two types of hydrogels, A1 and B1, which have lower C and MW values, resulting in a lower density. These hydrogels can endure temperatures of 37 °C and constant agitation at 500 rpm. In that case, it is expected that other hydrogels with higher C and MW values will also be able to endure these conditions due to their higher density [3,7].

After confirming that the PVA hydrogel in capsule form can store substances and withstand body temperatures under constant agitation, we obtained the results for the target substance of this study, which is the volume of glucose supported by two groups of hydrogels, A and B, that were previously dehydrated. Table 3 shows the glucose storage capacity of group A and B hydrogels. The results represent an average of five hydrogels for each PVA hydrogel formulation.

The maximum average volume supported by hydrogel A1 of group A was 42.10 ± 8.08 glucose units, equivalent to 421 µL. In comparison, hydrogel B1 of group B supported 44.90 ± 8.10 units, equivalent to 449 µL, with a conversion rate of 10 units to 100 µL. It is important to note that the hydrogels in group B are longer than those in group A, which affords them a greater storage capacity. However, as the C and MW increase, the difference in glucose volume between the hydrogels of both groups becomes shorter. This is because denser hydrogels have a lower storage capacity, but their ability to retain substances improves due to their strengthened structure [3,37].

As explained in the methodology, the glucose injection needle was inserted at the center of the hydrogel to maximize each hydrogel’s storage capacity. This ensured an even pressure distribution throughout the hydrogel’s walls. If the needle had been placed too close to the side walls, the hydrogel would have collapsed before reaching its total volume.

Understanding how substances are stored in hydrogels is crucial to predicting their release behavior over time [3]. This knowledge could help preserve cells encapsulated within the hydrogel by exchanging nutrients, but more reliable evidence is needed to confirm this [39].

### 2.4. Elongation at Breack

Table 4 displays the elongation at the break of PVA hydrogels with different C and MW. A total of 40 hydrogels was used, with 5 for each experiment. The hydrogel with MW2 (A3, A4 and B3, B4) exhibited a higher elongation at the break than those with MW1 (A1, A2 and B1, B2). This is because longer polymer chains can intertwine and form a more flexible and resistant network, allowing for greater deformation before breaking. In addition, the studied hydrogels have medium MWs, resulting in better overall mechanical properties characterized by high strength and toughness [40].

Regarding the hydrogel concentration, the higher-concentration hydrogels (A2, A4, and B2, B4) exhibit a greater elongation at break compared to the lower-concentration hydrogels (A1, A3, and B1, B3). This is because a higher concentration of PVA generally leads to a denser and more cross-linked polymeric network, thereby increasing the mechanical strength of the hydrogel [40]. This is also evident in the standard deviation, where there is a broader scatter of data points in the lower-concentration hydrogels.

As for the dimensions of the hydrogels, the smaller hydrogels (group A) demonstrate a lower elongation at break than the longer hydrogels (group B) because they have less material to distribute the stress. However, this is also influenced by the composition of the hydrogel and its manufacturing process, both of which are consistent in this case.

Elongation at break is crucial in applications where the hydrogel must withstand mechanical stresses, such as in scaffolds for tissue regeneration and sustained drug release [40].

### 2.5. Scanning Electron Microscopy (SEM) and Porosity Distribution

**Figure 2:**
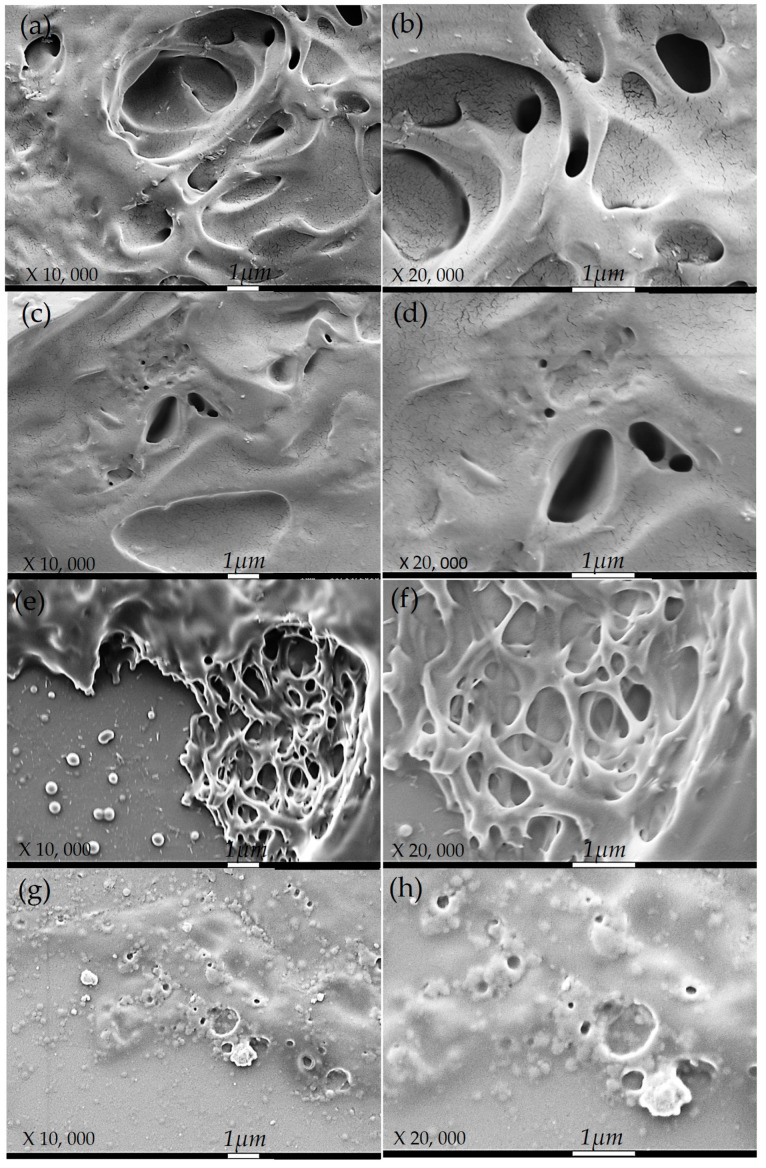
SEM images show more representatives of the group A hydrogels with resolutions ×10,000 and ×20,000, respectively: (A1) hydrogel (**a**,**b**), (A2) hydrogel (**c**,**d**), (A3) hydrogel (**e**,**f**), and (A4) hydrogel (**g**,**h**).

The formulation process for group A and B hydrogels is identical; the only distinction lies in their length. Consequently, Figure 2 displays the most representative SEM images of group A hydrogels, with two resolutions, ×10,000 and ×20,000, and varying C and MW. These images provide information on the structure and pore distribution.

The hydrogels labeled A1, which have a low C and MW of PVA, display a porous structure characterized by larger and less dense pores. This is due to the low C and MW, leading to a less dense polymeric network, allowing larger pores to form (see Figure 2a,b). The hydrogel A2, with a high PVA concentration and low MW, exhibits a structure with smaller and denser pores. Despite the low MW, the high PVA concentration increases the polymeric network’s density, reducing the pore size (Figure 2c,d). The A3 hydrogels with a low C and high MW exhibit a structure with intermediate-sized pores and a more uniform network. The high MW contributes to a better network formation. However, the low C limits the overall density (refer to Figure 2e,f). Finally, high-concentration and high MW hydrogels (A4) exhibit a very dense structure with small and uniform pores (Figure 2g,h). Due to their high C and MW, these hydrogels have a thick, uniform polymeric network with tiny, well-distributed pores. Some studies support this information [2,40].

**Figure 3:**
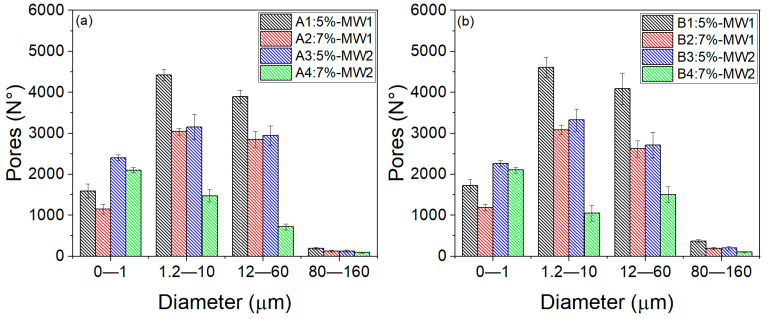
Histograms depict the porosity distribution (number of pores versus diameter) for the A1-A4 hydrogels (**a**) and B1-B4 hydrogels (**b**).

Image analysis can enhance our comprehension of how PVA concentration and MW impact the structure and characteristics of hydrogels. Altering the PVA concentration and MW can also assist us in understanding the release of glucose through the porosity of hydrogels. Figure 3 displays the distribution of the porosity, which represents the number of pores based on their diameters for the hydrogels in groups A and B.

The porosity distribution of the hydrogels in group A is similar to that of group B, with minor differences. This is because both groups were prepared using the same formulation. That is, the same C and MW used in group A were also used in the hydrogels of group B. The only distinction between the groups is the size of the hydrogels in group B, which could impact the release of glucose depending on the storage capacity of the hydrogel.

The hydrogels with a higher MW (A3, A4, B3, B4) have more pores from 0 to 1 µm than those with a lower MW (A1, A2, B1 and B2). Additionally, the hydrogels with a lower C for both MW values have a greater porosity than those with a higher C. Specifically, A1 has a greater porosity than A2, and B1 has a greater porosity than B2; similarly, A3 has a greater porosity than A4, and B3 has a greater porosity than B4 in the same range from 0 to 1 µm.

For PVA hydrogels with diameters of 1.2–10 µm, 12–60 µm, and 80–160 µm, the higher C hydrogels with both MW values (A2, A4, B2, B4) exhibit a lower porosity compared to the lower C hydrogels and both MW values (A1, A3, B1, B3). It should be noted that, although the lower C and lower MW hydrogels have higher porosities at different diameters, their standard deviation is higher due to non-uniform porosity distribution, specifically the hydrogels A1 and B1.

According to the behavior of the porosity distribution by changing the C and MW of PVA mentioned in previous paragraphs, PVA hydrogels with a low C and low MW have a porous structure with larger and less dense pores, resulting in a less dense polymeric network that allows the formation of larger pores. On the other hand, hydrogels with a high C and high MW show a very dense structure with small and uniform pores, resulting in a thick and uniform polymeric network with tiny and well-distributed pores.

A standardized preparation method can maintain a hydrogel’s porosity distribution, helping prevent significant variations in data, particularly in glucose release. However, the mechanical properties of an older hydrogel may change compared to a new one, leading to significant variability in the porosity distribution and glucose release behavior results [41].

### 2.6. Permeability Tests

This section illustrates the cumulative release, diffusion speed, and glucose flow from the inside to the outside of PVA hydrogel of all formulations for both A and B groups.

#### 2.6.1. Glucose Release

**Figure 4:**
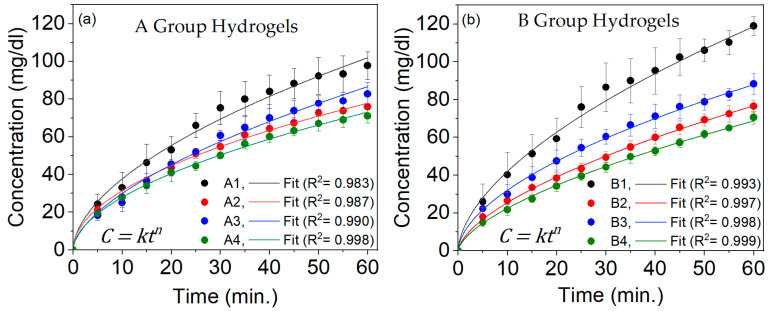
The curves showing the accumulation of glucose from inside to outside PVA hydrogels, presented as concentration versus time for group A (**a**) and group B (**b**) hydrogels.

The release of glucose through the pores of the hydrogels was analyzed using the Korsmeyer–Peppas model Equation (5), commonly used to describe drug release from PVA hydrogels. The fitted curve of the glucose release behavior shows a high coefficient of determination (R^2^) in Figure 4.

The hydrogels in group A had an *n* value of 0.5, while those in group B had an *n* value of 0.6. This means that the glucose release mechanism for group A hydrogels follows Fickian diffusion, while for group B hydrogels, it involves Fickian diffusion and the relaxation phenomenon [8].

Glucose release from inside to outside of the hydrogel could last more than 60 min, especially for the longest hydrogel. However, we decided to monitor the release for 60 min because we noticed a slight slowdown. We considered that the hydrogel would reach equilibrium later, where the concentrations inside and outside the hydrogel would equalize, and the diffusion would significantly slow down.

Glucose release from group A and B hydrogels started to be measured at minute 5. From 5 to 20 min, no differences were observed in the hydrogels of group A. This could indicate that glucose may start to exit through the largest pores in the four types of hydrogels (80–160 um), as they all have similar numbers of pores with slight variations due to C and MW, as shown in Figure 3a. From 25 to 60 min, statistically significant differences (*p*-value < 0.05) were observed between hydrogels A1 and (A2, A3, and A4), and A3 and A4. This difference is related to the distribution of porosity: at higher C and MW values, the structure is denser, resulting in smaller but more uniformly distributed pores.

In the comparison of hydrogels in group B, from 15 to 60 min, statistically significant differences (*p*-value < 0.05) were observed between B1 and (B2 and B4). Additionally, differences between B1 and B3 were observed from 25 to 60 min. Furthermore, from 45 to 60 min, differences were observed between B3 and (B2 and B4). As time progresses, differences between certain hydrogels become more apparent, which was initially obscured by the intersecting standard deviations of the hydrogels. The onset of glucose release from 0 to 10 min is linked to the larger pore distribution (80–160 µm) due to similar pore amounts with slight variations by C and MW, as depicted in Figure 3b.

The comparison between hydrogel groups A and B showed no differences between 5 and 10 min. However, statistically significant differences (*p*-value < 0.05) were observed between the following times:

- From 15 to 60 min between A1 and B4.
- From 20 to 60 min between A4 and B1.
- From 25 to 60 min between A2 and B1, A3 and B1, and A4 and B2.
- From 45 to 50 min between A3 and B4.

Furthermore, differences were observed between A1 and B1, as well as between A4 and B3, from 55 to 60 min. This glucose release behavior is related to the porosity distribution of each hydrogel type, as seen in Figure 3.

Although the glucose release behavior through the hydrogels of group B and group A is similar, differences in the release between the two groups are due to their length and the formation of the polymeric network along the material. These variations affect the hydrogel’s swilling ratio, glucose storage capacity, and mechanical properties, as evidenced in Table 2, Table 3 and Table 4.

After analyzing the release curves of hydrogels from groups A and B, we found that the distribution of pores may have a greater impact on the initial release (from 0 to 20 min) for pore sizes of 80–160 µm, as the porosity levels are similar. However, from 25 to 60 min, the porosity distribution in the 0–1, 1.2–10, and 12–60 µm pore size ranges may have a more significant influence, as indicated in Figure 3 by the noticeable difference in porosity levels for each hydrogel.

Understanding the release behavior of glucose from capsule-shaped PVA hydrogel is essential for several reasons. It enables the design of controlled release systems, ensuring that glucose is released at the right place and time. We can only write these general statements based on glucose because every drug has a different structure. This understanding also helps fine-tune hydrogel properties such as porosity, C, and MW to achieve the desired release profile. Additionally, it reduces glucose wastage by allowing a better control over the amount released, thus avoiding overdoses or premature release. Furthermore, it ensures that the glucose remains stable and avoids degradation.

#### 2.6.2. Diffusion Speed

**Figure 5:**
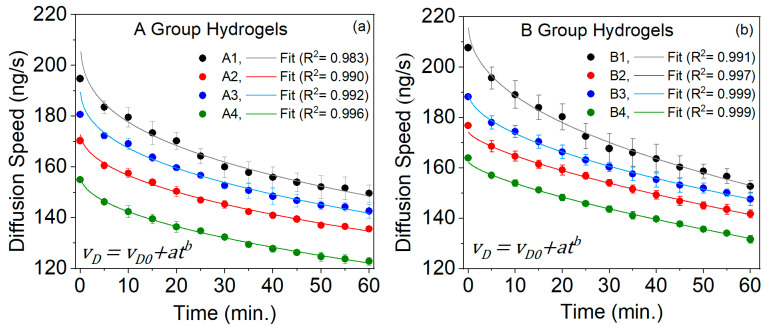
Diffusion speed $\left(v_{D}\right)$ versus sampling time for the group A (**a**) and group B (**b**) PVA hydrogels.

Figure 5 shows the behavior of the diffusion speed ${(v}_{D})$ curves for glucose through the two groups of hydrogels, A and B. The $v_{D}$ data are fitted to a power (allometric) equation with a high R^2^ value. $v_{D}$ decreases with the increase in the sampling time. This is because, as glucose diffuses, the concentration in the hydrogel decreases, reducing the concentration gradient that drives diffusion Equation (6).

The $v_{D}$ of glucose through PVA hydrogels is the speed at which glucose molecules move through this matrix. When comparing the $v_{D}$ between hydrogels in group A, we observed statistically significant differences (*p*-value < 0.05) from 0 to 60 min, except for the interval from 35 to 60 min for hydrogels A1 and A3, because their $v_{D}$ values are closer, so their standard deviations intersect and follow the order of hydrogels from the highest to the lowest: A1 > A3 > A2 > A4. The decrease in $v_{D}$ over longer intervals occurs as glucose diffuses out of the hydrogel, reducing the amount of glucose inside it. Several factors influenced these processes:

(a) Pore size: where larger pores (A1 > A3 > A2 > A4) allowed a faster diffusion (refer to Figure 3).
(b) Concentration gradient: the higher gradient generally resulted in a higher diffusion speed (A1 > A3 > A2 > A4); this is a consequence of item (a).
(c) Temperature: Higher temperatures cause molecules to move faster, increasing the diffusion speed. The hydrogels were exposed to 37 °C, allowing for a better glucose molecule diffusion.
(d) Molecule size: Smaller molecules diffuse through the hydrogels faster than larger ones. The glucose molecule is of the nanometric order (approximately 0.8 nm), which favors its diffusion.

There are significant statistical differences (*p*-value < 0.05) between the hydrogels of group B, except from 30 to 45 min of hydrogel B1 versus B3 due to their point spread. The $v_{D}$ from the highest to the lowest for each hydrogel was B1 > B3 > B2 > B4. The same factors influence this sequence as those mentioned for the hydrogels of group A. However, these hydrogels are longer than group A, which might affect the $v_{D}$. Therefore, in the next paragraph, we compare the $v_{D}$ for the two hydrogel groups, A and B.

When comparing the $v_{D}$ between hydrogel groups A and B from 0 to 60 min, the following statistically significant (*p*-value < 0.05) differences were observed:

- A2 had differences compared to B1 and B2.
- A3 had differences compared to B1 and B2.
- A4 had differences compared to B1, B2, B3, and B4.

Furthermore, differences were observed between A1 and B1, B2, and B4 from 0 to 60 min, except from 30 to 50 min, due to the point dispersion, where standard deviations overlapped.

The hydrogels in group B are longer than those in group A, which enables them to support more glucose. This difference becomes significant in the $v_{D}$. It is theorized that, in longer hydrogels, the concentration gradient can decrease as the substance diffuses, slowing down the $v_{D}$ in the more distant sections. However, despite this, hydrogels from group B exhibited a higher $v_{D}$. This could be attributed to the fact that glucose diffusion through these hydrogels occurred from the center to the outside because the concentration of glucose was higher in the center (the cylindrical shape of the hydrogel) than in the distant parts of the hydrogel (hemispherical ends of the hydrogel).

In conclusion, although the length of the hydrogel can impact the $v_{D}$, other factors, such as the concentration gradient, hydrogel structure, and chemical interactions, are equally important.

#### 2.6.3. Diffusion Flow

The diffusion flux (*J*) is related to $v_{D}$, which represents the amount of glucose moving through the hydrogel per unit area and time, as shown in Equation (8). The negative sign in the *J* equation indicates that the substance moves in the direction that reduces the concentration gradient, ensuring that the flux is positive in that direction.

**Figure 6:**
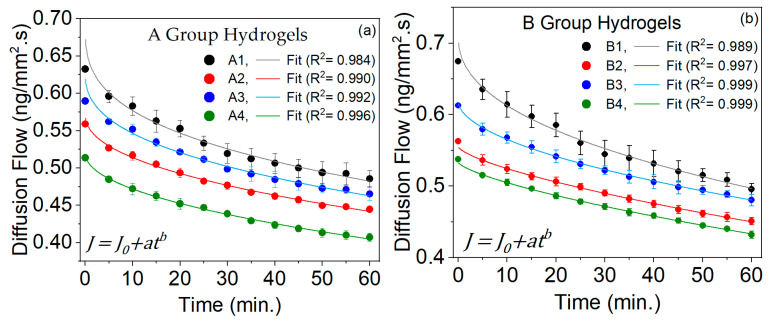
Diffusion flow versus sampling time for the group A (**a**) and group B (**b**) PVA hydrogels.

Figure 6 illustrates the *J* behavior of glucose through the PVA hydrogels of groups A and B. This behavior fits very well with a power (allometric) equation, showing a high R^2^.

A statistically significant difference (*p*-value < 0.05) was observed between all hydrogels in group A when comparing them from 0 to 60 min, except from 35 to 60 min for hydrogels A1 and A3. This exception occurred because their *J* is closer, leading to their standard deviation crossing. Similarly, the hydrogels in group B also exhibited statistically significant differences (*p*-value < 0.05) when compared to each other, except from 35 to 60 min for hydrogels B1 and B3, due to the same reason that occurred between hydrogels A1 and A3.

When comparing the two groups of hydrogels A and B, we found statistically significant differences (*p*-value < 0.05), as follows:

- From 5 to 10 min, differences were observed between A3 and B2.
- From 5 to 25 min, differences were observed between A1 and B1.
- From 5 to 60 min, differences were observed between A1 and (A2 and A4), A3 and B1, A3 and B4, and A4 and (B1, B2, B3 and B4).

The distribution of porosity in PVA hydrogels plays a crucial role in facilitating the flow of glucose. Below are some key reasons that we believe are critical for achieving controlled dispersion in the results for each type of hydrogel:

(a) Using a standardized construction methodology (see Section 4.1), the pores of adequate size were uniformly distributed throughout each hydrogel depending on the C and MW, facilitating the movement of glucose molecules through the hydrogel. Larger and well-connected pores allow for a faster and more efficient diffusion.
(b) A reasonable amount of porosity was obtained, which increased the contact surface between the hydrogel and glucose, improving the interaction and, therefore, diffusion.
(c) The porosity distribution helped reduce the resistance to glucose flow, allowing for a less constrained and faster movement through the hydrogel.
(d) The porosity distribution obtained for each hydrogel allowed for balancing glucose retention and controlled release. It is worth mentioning that, in this study, the sampling time for glucose release was up to 60 min. However, a more extended release could be obtained until the maximum glucose release.

In summary, a well-designed porosity distribution according to the C and MW in PVA hydrogels is essential to optimize the diffusion of glucose or other substances, improving the process’s efficiency and effectiveness.

### 2.7. Cell Proliferation

To confirm that the biomaterial did not inhibit cell proliferation or induce cell death, an experiment was conducted to determine its biocompatibility.

The cell line in direct contact with the 6 A1 hydrogels does not interfere with cell proliferation in culture (*p* = 0.467). The slight variations in the number of cells in each hydrogel could be attributed to differences in porosity distribution. The cell growth response and viability at 48 h in contact with the hydrogels was similar to that of the control (4.5 × 10^5^ cells/mL, 98.5% vs. 4.8 × 10^5^ cells/mL, 99.7% viability), thus demonstrating biocompatibility.

**Figure 7:**
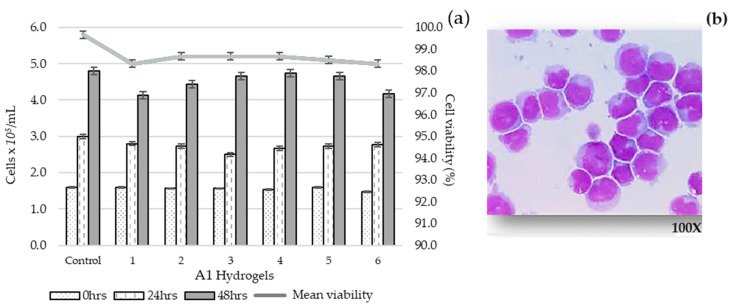
Cell proliferation and viability of the control sample (cell line in culture) and the cell line in contact with the 6 A1 hydrogels for 24 and 48 h with MTS Cell proliferation assay (**a**) and cell morphology (Wright stain) observed in cytospin (100×) (**b**). The cell count was performed in triplicate for each hydrogel.

In Figure 7a, the graph columns represent cell proliferation at 0, 24, and 48 h, proving no inhibition in cell proliferation or death due to cell–hydrogel contact. Figure 7b corresponds to a cytospin that verifies the characteristic morphology of the cell line used (SUP-B15, ATCC CRL-1929, Manassas, VA, USA).

Based on the standardized preparation and mechanical properties of hydrogels from groups A and B and the encouraging cell growth response, the project’s next phase involves using various cell lines in direct contact with and within the hydrogels to assess their survival and proliferation rates.

## 3. Conclusions

The research found that low C and MW PVA capsule-shaped hydrogels have a wide range of porosity. Specifically, in the range of 0–1 µm, A1 > A2 and A3 > A4, and in the range of 1.2–160 µm, A1 > A3 > A2 > A4. This behavior is similar to the hydrogels of group B, with the only difference being their larger size. The porosity distribution of hydrogels A1 and B1 is wider. Still, these have a greater margin of error, and hydrogels with a higher C and MW have a more uniform porosity distribution, allowing for a more controlled release. Due to their wide porosity distribution, hydrogels A1 and B1 showed a greater glucose storage capacity, with B1 being greater than A1 due to its larger size. All hydrogels remained stable at 37 °C and under stirring at 500 rpm without degrading.

Statistically significant differences (*p*-value < 0.05) were found in the cumulative glucose concentration, diffusion speed, and diffusion flux between the hydrogels, particularly between A1 > A4, B1 > B4, and B1 > A1 over 60 min. Group B’s hydrogels were larger than group A’s, supporting a higher glucose content. The C and MW influenced the diffusion of glucose, allowing for a rapid or slow diffusion regardless of the size of the hydrogel, making them suitable for various applications such as releasing hydrophilic substances or preserving cells (e.g., SUP-B15 cell line) due to their biocompatibility.

The biocompatibility experiment results show that the contact between cells and the hydrogel did not have an anti-proliferative effect or impact cell viability.

## 4. Materials and Methods

### 4.1. Preparation of PVA Hydrogels

We followed a two-step process to prepare the capsule-shaped hydrogels of two sizes, two Cs and two MWs of PVA. Part of the methodology was adapted from a study [2].

Step 1: PVA gel

To prepare the gel, PVA powder with a 99% hydrolysis degree (Sigma Aldrich, Saint Louis, MO, USA) of MW1 = 85,000–124,000 and MW2 = 146,000–186,000, with concentrations of C1 = 5% and C2 = 7%, was used along with ultrapure water of MilliQ grade (Merck Millipore, Darmstadt, Germany). We used the following equation to achieve the desired PVA concentration:

(1) $$W_{PVA}\left(g\right)=\left[PVA\;concentration\;\left(\%\right)\times H_{2}O\;volume\;(mL)\right]/100\%$$

To prepare the gel for C1, MW1, and MW2 of PVA, 80 mL of ultrapure water and 4 g of PVA powder were mixed. For C2, MW1, and MW2 of PVA, a mixture of 80 mL of pure water and 5.6 g of PVA powder was used. The mixtures were heated from 20 °C to 85 °C for around 90 min while stirring with a magnetic bar to ensure a uniform solution. After heating, the gel was allowed to cool down to room temperature (20 °C) to remove any air bubbles.

Step 2: PVA cryogel

**Figure 8:**
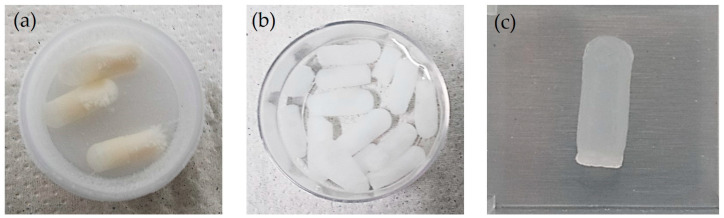
(**a**) PVA hydrogels in the capsule molds after the fourth frozen cycle, (**b**) PVA hydrogels immersed in ultrapure water four days after their preparation, and (**c**) PVA hydrogel at room temperature (20 °C) after the fourth freezing cycle.

The gel obtained in the first step was poured into capsule-shaped molds of two sizes: L1 = 10 mm (300 µL PVA gel) and L2 = 20 mm (600 µL PVA gel), both with the same diameter of 6 mm, which had been previously sterilized. Subsequently, the molds filled with PVA gel were subjected to a freezing process at −80 °C using an ultra-freezer (Kaltis GV039M, Shanghai, China) for 20 min and then thawed at room temperature (20 °C) for 40 min in four cycles. Figure 8a shows the frozen hydrogels in the capsule molds after the fourth cycle. In contrast, Figure 8b displays the hydrogels removed from their molds four days after their preparation, which is why it is denser than the hydrogel in Figure 8c, which is the hydrogel thawed at room temperature after the fourth freezing cycle.

Finally, 32 capsule-shaped hydrogels, consisting of 16 L1 hydrogels and 16 L2 hydrogels, were obtained. Table 5 displays the hydrogels labeled by concentration, molecular weight, and size.

The group A and B hydrogels are L1 = 10 mm and L2 = 20 mm long, respectively; four capsules were used for each C and MW. One side becomes more flattened after the hydrogels are removed from their molds, as depicted in Figure 8c. The hydrogels are placed in ultrapure water to maintain their viability for several days, as illustrated in Figure 8b.

The same methodology (standardization) was used to prepare PVA hydrogels for reproducibility and comparability.

(a) The Cs and MWs of PVA were carefully selected without using chemical cross-linking agents for biocompatibility testing.
(b) The PVA powder and ultrapure water were mixed according to a specific procedure, including heating and stirring.
(c) The optimal stirring temperature and frequency for gel formation were identified.
(d) The adequate temperature and timing of the freeze/thaw cycles were determined to achieve a homogeneous porosity distribution for each hydrogel formulation.
(e) The precise volume of PVA gel was poured into gelatin capsule molds corresponding to lengths of 10 and 20 mm.
(f) All steps were thoroughly documented for future reference.

The preparation of hydrogels can vary depending on the objective. This study was conducted to prepare hydrogels for controlled glucose release and cellular biocompatibility. Therefore, standardizing the methodology for each type of hydrogel helps minimize variations between hydrogel formulations and ensures consistent results, which are crucial for biomedical and tissue engineering applications.

### 4.2. Dehydration

A closed system at 20 °C containing silica gel granules and a device that the hydrogel released as a water filter was used for the controlled dehydration process. The function of the silica gel is to absorb the water in the hydrogel. With the help of this system, it was possible to monitor the hydrogel’s dehydration by measuring their length and water mass loss every 15 min for 1 hour for group A and every hour for 24 h for group B group. This mechanism was used before hydrogels were filled with glucose to support a greater volume.

The following equation calculates the water mass loss in percentage (*W_L_*) for all PVA hydrogel formulations.

(2) $$W_{L}\left(\%\right)=\frac{W_{1}-W_{2}}{W_{1}}\times 100\%$$

where *W*_1_ and *W*_2_ are the respective weights of the hydrogel before and after dehydration; each weight was measured four times to obtain an average value.

### 4.3. Swelling Ratio Determination

To determine the hydrogels’ swelling ratio (*W_s_*), we injected 150 µL of ultrapure water into the L1 hydrogels and 300 µL into the L2 hydrogels. We then wiped off any excess water before measuring their weight. After that, the samples were freeze-dried and incubated at room temperature (20 °C) and 50.4% relative humidity for 12 h for L1 hydrogels and 20 h for L2 hydrogels. Following incubation, their weight was measured again, and the swelling ratio was calculated using Equation (3).

(3) $$W_{s}\left(\%\right)=\frac{W_{3}-W_{2}}{W_{2}}\times 100\%$$

*W*_3_ represents the sample weight in a swollen state, and *W*_2_ represents the sample weight in a dry state. Each test was repeated four times to obtain an average value. This experiment was conducted according to M. Huang et al. and E. Rosa et al. [5,42].

### 4.4. Storage Capacity and Thermal Resistance

The PVA hydrogels were tested for storage capacity and thermal resistance using milk and green vegetable dye. This ensured the mixture filled the hydrogel and released it from the inside to the outside. Glucose was not used initially in this test due to its transparency; so, milk, with a molecule diameter similar to that of glucose, was used instead.

**Figure 9:**
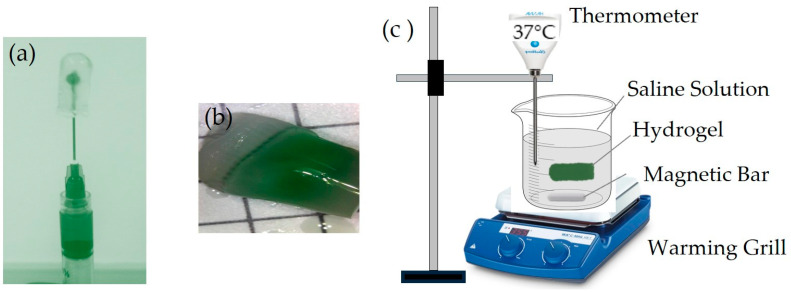
(**a**) Injection of the milk mixture with green vegetable dye into the PVA hydrogel using an insulin syringe, (**b**) PVA hydrogel sealed with a toothed clamp to prevent any leakage due injection of the mixture, and (**c**) proofs of PVA hydrogel degradation and release of the mixture in saline solution to 37 °C and constant stirring of 500 rpm.

To test the storage capacity, an insulin syringe was used to inject a maximum volume of mixture into the center of the hydrogel (Figure 9a). The hydrogel was then sealed with a toothed clamp subjected to a force of 20 kg/cm^2^ to prevent leakage (Figure 9b). The sealed hydrogel was then placed in 20 mL of saline solution at 37 °C and constantly stirred at 500 rpm to assess the release of the mixture for 60 min (Figure 9c).

Tests were only conducted on four A1-type and four B1-type hydrogels to ensure the proper filling and release of the mixture. This allowed for subsequent tests with greater reliability using glucose, the study’s target substance, in all hydrogel formulations.

In the glucose filling tests for all PVA hydrogel formulations, the same procedure, including sealing the injection hole, was used for the milk and green vegetable dye mixture. A similar procedure was followed to test glucose release from inside to outside the hydrogel, but a glucometer was used to detect cumulative glucose release. This procedure is described in more detail in Section 4.6 on permeability tests.

### 4.5. Elongation at Break

**Figure 10:**
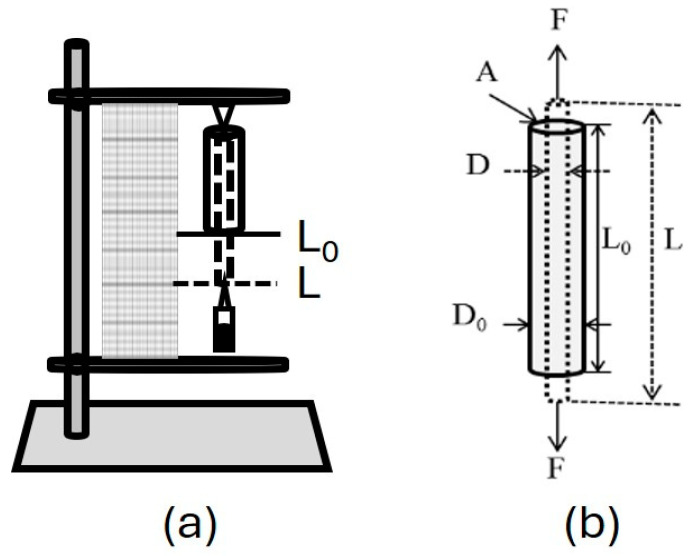
Schematic representation of the device used for uniaxial tensile testing of PVA hydrogels (**a**) and the uniaxial deformation of the PVA hydrogel under tensile forces (**b**).

Figure 10 shows that the PVA hydrogels underwent a tensile test using a system designed by the Research and Technological Development Unit (UIDT) of the General Hospital of Mexico “Dr. Eduardo Liceaga”.

The elongation at the break of PVA hydrogel measures the material’s ability to stretch before it breaks. This property is determined using the elastic or Young’s modulus equation, represented in Equation (4).

(4) E=dσ/dε

where dσ is the longitudinal stress and dε is longitudinal deformation. This system was chosen because the samples are macro-sized, which helps to avoid significant errors. Four experiments were conducted for each hydrogel.

### 4.6. Scanning Electron Microscopy (SEM) and Porosity Distribution

The morphological nature of the PVA hydrogels in this study was identified by obtaining scanning electron microscopy (SEM) images using a (Quanta 3D FEG, Santa Clara, CA, USA) at an acceleration voltage of 2 kV. Before the SEM analysis, the samples were gold-coated and allowed to dry on filter paper at room temperature for 15 min.

Eight images of 50 µm thick slices were obtained from sections of four hydrogels of the same type to obtain a more robust porosity distribution.

The SEM images were converted to 8-bit format and then binarized using the ImageJ software to analyze the porosity distribution of PVA hydrogels. After binarization, the MATLAB R2021b-academic use software applied filters to the images to enhance the resolution.

### 4.7. Permeability Tests

Fifty units (ten units equals 100 µL) of (10% dextrose, Baxter, Mexico City, Mexico) glucose were injected into all the previously dehydrated PVA hydrogels [43]. The amount of glucose absorbed by each hydrogel depended on the C and MW [37]. These hydrogels were then immersed in 20 mL of saline solution (0.9% sodium chloride, Baxter, Mexico City, Mexico) and stirred continuously at 500 rpm at 37 °C. Using a glucometer (Accu-Chek, Mexico City, Mexico), the glucose concentration released into the saline solution was measured at 5 min intervals for one hour.

Glucose was selected due to its biocompatibility, non-toxic nature, and suitable molecular size for efficient diffusion through the PVA hydrogel matrix, allowing for controlled and sustained release. Specific sensors (glucometer) could also accurately monitor its concentration and release. Moreover, glucose is an essential energy source for the body’s cells. It can have various medical applications, such as in diabetes treatment or as a nutrient exchange for cells within the hydrogel [19,20].

Saline solution was chosen as the medium for glucose release due to its pH, which is generally similar to that of the human body, making it compatible with body tissues and fluids.

#### Glucose Release Model

The nonlinear regression model Korsmeyer–Peppas was used to perform the curve fit of glucose release [44], since it is often used to describe the release of substances through PVA hydrogels. This is because of its versatility, simplicity, and ability to fit the data well, providing an accurate characterization. It helps generate useful parameters such as *n* and *k* to understand the release mechanisms and their applicability to a release system. When *n* equals 0.5, the substance release mechanism is Fickian diffusion. If *n* is between 0.5 and 1, it involves both Fickian diffusion and relaxation phenomenon [8].

(5) $${C=M}_{t}/M_{\infty }=k\cdot t^{n}$$

where $M_{t}/M_{\infty }$ represents the fractional permeated glucose, *t* is the time, *k* is the transport constant (dimension of time^−1^), and *n* is the transport exponent (dimensionless). The release constant *k* provides mostly information on the glucose formulation, whereas *n* is important since it relates to the glucose release mechanism.

### 4.8. Diffusion Speed

The hydrogels utilized in this study exhibit a non-stationary glucose release mechanism. Therefore, the speed of glucose diffusion is directly linked to the difference in concentration at any given moment and is not dependent on time. For this reason, Fick’s second law is employed to calculate the glucose diffusion speed.

(6) $$v_{D}=D\frac{A_{h}\left(C1-C2\right)}{r}$$

where D is the glucose diffusion coefficient to 25 °C, which is $6.76\times 10^{-4}\;{mm}^{2}s^{-1}$ [45]; *A_h_* is the surface area of the hydrogel; C1 is the highest concentration inside the hydrogel; C2 is the region (saline solution) of the lowest concentration; and *r* is the radius of the hydrogel, representing the path for glucose diffusion from the center to the outside.

Since the hydrogel has a capsule shape (spherocylinder), the following geometric equation was used to calculate its surface area.

(7) $$A_{h}=A_{c}+A_{he}=2\pi rh+2\left(2\pi r^{2}\right)$$

where *A_c_* is the cylinder’s curved surface area, *A_he_* is the hemisphere’s curved surface area, and *r* is the radius of the hydrogel.

To determine the thickness of empty hydrogels and hydrogels filled with glucose, we utilized photographs of the hydrogels and the Adobe Photoshop Express 2022 photo editing tool. This method allowed us to achieve greater accuracy through the use of pixels. The dimensions of the hydrogels are shown in Table 6.

The length of the hydrogel remains constant regardless of the amount of glucose present, as it is distributed more evenly in the central part of the capsule, which has a cylindrical shape. As a result, we present the diameter of the hydrogel (*d*), the surface area (*A_h_*), and the volume with and without glucose for comparison.

### 4.9. Diffusion Flow

From the diffusion speed, we can calculate the diffusion flow (*J*):

(8) $$J=-\frac{v_{D}}{A_{h}}.$$

*J* was calculated using the Section diffusion speed method, where *A_h_* is the surface area of the hydrogel and $v_{D}$ is the diffusion speed. The negative sign indicates that glucose diffuses towards a decreasing concentration gradient. This means that the glucose molecules move from a higher concentration (hydrogel) to a region of lower concentration (saline solution), and the concentration decreases in the direction of flow.

The analysis of *J* was plotted as a positive value to facilitate the interpretation of the data and comparison between the different experiments, i.e., between the various hydrogels. A positive *J* indicates the amount of glucose moving through the PVA hydrogel.

### 4.10. Sterilization Process

To ensure a sterile environment for preparing the PVA gel and hydrogel, we followed these steps:

(a) The material was sterilized using a Steri-Vac^®^ 8XL sterilization chamber (3M Health Care, St. Paul, MN, USA).
(b) We kept the entire workspace clean with bleach.
(c) We cleaned the laminar flow hood (Biobase, BBS-SDC, Jinan, China) inside and out with benzalkonium chloride.
(d) We filled the laminar flow hood with equipment and materials needed to prepare the PVA hydrogel.
(e) We irradiated the interior of the laminar flow hood with UV rays for 15 min.
(f) We wore appropriate clothing and applied benzal to gloves to minimize contamination.
(g) We prepared the PVA gel and poured it into molds inside the laminar flow hood.
(h) We covered the molds with sterilized aluminum foil before transferring to the freezer.
(i) We used aerosols to eliminate viruses and bacteria in the path from the laminar flow hood to the freezer.
(j) We thawed and checked the hydrogel for sterility using a Falcon-type tube and microbiological growth juice.

These hydrogels can serve as suitable environments for optimal growth and development of cells within a living being without causing any adverse reactions or rejection.

### 4.11. Cell Proliferation Test

**Figure 11:**
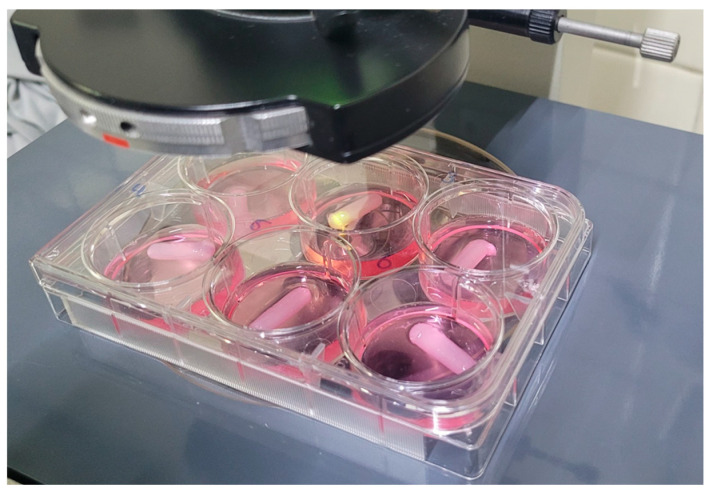
Microplate with A1 hydrogels and cell line SUP-B15 in IMDM medium with 20% BFS.

After the PVA hydrogels were sterilized, a cell proliferation test was conducted using the SUP-B15 (ATCC CRL-1929) cell line in an IMDM medium with 20% fetal bovine serum (FBS) [46] to assess cell proliferation and survival in direct contact with PVA hydrogels [47,48]. The SUP-B15 cell line was placed in direct contact with 6 A1 hydrogels evenly distributed in a multiwell dish, as illustrated in Figure 11. The multiwell dish was then incubated at 37 °C with 5% CO_2_ for 24 and 48 h.

The hematological cell line SUP-B15, catalog number CRL-1929, was acquired directly from American Type Culture Collection (ATCC, Manassas, VA, USA) [46].

We utilized CellTiter 96^®^ AQueous Non-Radioactive Cell Proliferation Assay (MTS) (PROMEGA, Madison, WI, USA) cell proliferation assay kit to measure viable cells, which is recognized for its precise quantification of viable cells [48].

To assess cell viability and proliferation, 100 µL of the cell suspension from each condition was mixed with 20 µL of a solution containing MTS and PMS in a 20:1 ratio. This mixture was placed in a 96-well plate (Corning, New York, NY, USA) and incubated for 2 h at 37 °C in a 5% CO_2_ atmosphere to allow the production of Formazan. Cell viability was measured using an iMark Microplate Absorbance Reader (Bio-Rad, Lasec, Cape Town, South Africa) to assess the absorbance of Formazan at 490 nm, which is directly proportional to the number of living cells. All measurements were carried out in triplicate.

### 4.12. Statistical Tests

The estimated marginal means method was used with Bonferroni adjustment to compare Generalized Linear Models. The hydrogels of group A, group B, and both groups at different monitoring times (0, 5, 10, 15, 20, 25, 30, 35, 40, 45, 50, 55, and 60 min) were compared for glucose concentration, diffusion speed, and diffusion flow. The analysis showed statistically significant differences with a *p*-value < 0.05. We conducted this statistical analysis using SPSS version 25.

## Figures and Tables

**Table 1:** The percentage of water mass loss (*W_L_*) in all PVA hydrogel formulations during dehydration.

Hydrogels (A Group)	*W_L_* (%)	Hydrogels (B Group)	*W_L_* (%)
A1	7.79 ±1.72	B1	10.6 ± 0.55
A2	4.23 ± 2.17	B2	6.80 ± 2.80
A3	6.25 ± 1.12	B3	7.46 ± 1.02
A4	3.94 ± 2.11	B4	3.98 ± 1.54

**Table 2:** Results of the swelling ratio ($W_{s}$) in all PVA hydrogel formulations.

Hydrogels (A Group)	$W_{s}$ (%)	Hydrogels (B Group)	$W_{s}$ (%)
A1	121 ±25	B1	141 ± 13
A2	96 ± 7	B2	110 ± 12
A3	115 ± 17	B3	130 ± 9
A4	103 ± 4	B4	115 ± 6

**Table 3:** The volume of glucose supported by the hydrogels of groups A and B.

Hydrogels (A Group)	Volume (Units)	Hydrogels (B Group)	Volume (Units)
A1	42.10 ± 8.08	B1	44.90 ± 8.10
A2	37.20 ± 8.06	B2	38.50 ± 8.14
A3	39.25 ± 6.08	B3	40.80 ± 6.80
A4	34.15 ± 6.05	B4	35.85 ± 6.10

**Table 4:** Elongation at break of the hydrogel groups A and B.

Hydrogels (Group A)	Elongation at Break (%)	Hydrogels (Group B)	Elongation at Break (%)
A1	151 ± 17	B1	153 ± 21
A2	161 ± 5	B2	164 ± 8
A3	156 ± 4	B3	158 ± 10
A4	165 ± 7	B4	168 ± 18

**Table 5:** Formulations of PVA hydrogels using two concentrations (Cs) and two molecular weights (MWs). The number of hydrogels prepared is indicated in parentheses.

Group	Hydrogels (N°)	C	MW
A (L1 = 10 mm)	A1 (4)	C1 = 5%	MW1 = 85,000–124,000
	A2 (4)	C2 = 7%	MW1= 85,000–124,000
	A3 (4)	C1 = 5%	MW2 = 146,000–186,000
	A4 (4)	C2 = 7%	MW2 = 146,000–186,000
B (L2 = 20 mm)	B1 (4)	C1 = 5%	MW1 = 85,000–124,000
	B2 (4)	C2 = 7%	MW2 = 85,000–124,000
	B3(4)	C1 = 5%	MW1 = 146,000–186,000
	B4 (4)	C2 = 7%	MW2 = 146,000–186,000

**Table 6:** Dimensions of the hydrogels. Each data point is an average of four hydrogels.

Hydrogels	Length (mm) (Empty = Glucose)	*d* (mm) (Empty/Glucose)	*A_h_* (mm^2^) (Empty/Glucose)	Volume (mm^3^) (Empty/Glucose)
A1	9.98 ± 0.15	5.99 ± 0.02/8.10 ± 0.16	187.87 ± 0.82/307.99 ± 10.03	225.02 ± 1.54/484.78 ± 24.35
A2	9.97 ± 0.04	5.96 ± 0.04/8.05 ± 0.13	186.24 ± 6.85/304.78 ± 8.21	225.02 ± 3.86/476.92 ± 17.04
A3	9.95 ± 0.09	5.98 ± 0.14/8.08 ± 0.11	187.80 ± 2.07/306.38 ± 7.06	222.00 ± 12.90/480.81 ± 19.84
A4	9.94 ± 0.04	5.95 ± 0.09/8.00 ± 0.10	186.01 ± 4.30/301.62 ± 6.28	221.61 ± 7.95/469.30 ± 15.08
B1	20.08 ± 0.19	6.00 ± 0.10/8.10 ± 0.10	188.43 ± 7.11/307.94 ± 6.35	226.21 ± 13.34/484.54 ± 15.40
B2	19.88 ± 0.13	5.98 ± 0.04/8.07 ± 0.09	187.25 ± 2.16/305.72 ± 5.90	223.87 ± 4.03/479.15 ± 14.28
B3	19.85 ± 0.11	5.98 ± 0.08/8.09 ± 0.15	187.26 ± 4.17/307.19 ± 9.62	223.93 ± 7.80/482.82 ± 23.34
B4	19.82 ± 0.11	5.96 ± 0.03/8.05 ± 0.09	186.37 ± 1.34/304.77 ± 5.89	222.22 ± 2.50/476.86 ± 14.16

## Data Availability

The original contributions presented in the study are included in the article; further inquiries can be directed to the corresponding author.
